# Current Evidence on the Potential Role of Endothelial *SHP-1* in Pulmonary Vascular Remodeling Associated With Pulmonary Hypertension

**DOI:** 10.31083/RCM39059

**Published:** 2026-01-13

**Authors:** Xinting Zhang, Jiao Yang, Zeyuan Yang, Ting Liu, Bingqian Zeng, Mingxi Ma, Ying Liu, Shuanglan Xu, Xiqian Xing

**Affiliations:** ^1^Department of Pulmonary and Critical Care Medicine, The Affiliated Hospital of Yunnan University, 650021 Kunming, Yunnan, China; ^2^College of Clinical Medicine, Dali University, 671003 Dali, Yunnan, China; ^3^Department of Pulmonary and Critical Care Medicine, First Affiliated Hospital of Kunming Medical University, 650032 Kunming, Yunnan, China; ^4^State Key Laboratory of Primate Biomedical Research, 650500 Kunming, Yunnan, China; ^5^Institute of Primate Translational Medicine, Kunming University of Sciences and Technology, 650500 Kunming, Yunnan, China; ^6^Key Laboratory of Respiratory Disease Research of Department of Education of Yunnan Province, 650021 Kunming, Yunnan, China

**Keywords:** pulmonary hypertension, *SHP-1*, endothelial cells, vascular remodelling

## Abstract

Pulmonary hypertension (PH) is characterized by an abnormally high pressure within the pulmonary arteries, which can be attributed to various factors. Severe diseases affecting pulmonary vessels may result in heart failure and potentially lead to death; these conditions are linked to significant mortality and unfavorable outcomes. Approximately 1% of adults worldwide have PH, and this condition may affect up to 10% of people older than 65 years. Currently, the mechanisms involved in the development of PH are not fully known and are thought to result from multiple coordinated factors. This lack of understanding remains a bottleneck in clinical practice. Numerous studies have confirmed that pulmonary artery endothelial cell (PAEC) dysfunction plays an important role in occlusive pulmonary vascular remodeling and the pathogenesis of PH. Src homology region 2 domain-containing phosphatase-1 (*SHP-1*) is a regulatory molecule that negatively modulates various cellular mediators and growth factors, primarily playing a negative regulatory role in signal transduction pathways. This review mainly presents an in-depth exploration of the key signaling pathways through which *SHP-1* regulates the expression of endothelial cells (ECs), thereby influencing various physiological functions, including proliferation, migration, oxidative stress, angiogenesis, apoptosis, autophagy, the inflammatory response, and vascular permeability. Furthermore, the potential mechanisms through which endothelial *SHP-1* plays a role in pulmonary vascular remodeling in PH are discussed. These findings underscore *SHP-1* as an encouraging therapeutic target for preventing and managing PH.

## 1. Introduction

Pulmonary hypertension refers to a disease that involves increased pulmonary vascular resistance. The pathological process of PH is characterized by pulmonary vascular remodelling, which involves excessive proliferation of pulmonary artery smooth muscle cells (PASMCs) and pulmonary artery endothelial cells (PAECs), distal pulmonary artery muscularization, vascular occlusion, plexiform lesions, and abnormal accumulation of inflammatory cells [1, 2, 3, 4]. Alterations in pulmonary vascular tone and remodelling contribute to a progressive increase in pulmonary vascular resistance, ultimately culminating in a spectrum of clinical syndromes associated with right heart failure and, in severe cases, death [5]. The diagnostic criteria for haemodynamics are outlined as follows: A mean pulmonary artery pressure (mPAP) of ≥20 mmHg, as assessed through right heart catheterization, indicates the presence of pulmonary hypertension (PH) under resting conditions at sea level [6]. One percent of adults across the globe are afflicted with PH, whereas its prevalence can reach 10% among individuals older than 65 years [7]. Currently, several drugs with vasodilatory effects, such as endothelin, nitric oxide, and prostacyclin, have been successfully developed to treat PH [6, 8].

Recent research has shown that among individuals undergoing standard treatment for PH, the use of sotatercept results in decreased pulmonary vascular resistance and increased haemodynamic metrics and exercise ability, as evaluated using the 6-minute walk test [9, 10]. These findings provide a new potential treatment strategy for PH [11]. Although current treatment methods can improve the quality of life of patients, none are curative, which presents a significant challenge in clinical practice [12]. In the early stages of PH development, EC injury and apoptosis are predominant [13, 14, 15]. Conversely, in the later stages of PH, EC overproliferation and antiapoptotic mechanisms prevail, resulting in significant vascular remodelling [16]. Recent studies have shown that endothelial cell dysfunction, injury, and immune‒inflammatory responses, along with metabolic abnormalities, epigenetic changes, endothelial‒mesenchymal transition (*EndMT*), and the release of growth factors and chemokines from endothelial cells (ECs), induce the proliferation of SMCs and play a role in the structural changes associated with pulmonary vascular remodelling. This process increases pulmonary artery pressure and pulmonary vascular resistance [17].

Src homology region 2 protein tyrosine phosphatase-1 (*SHP-1*), which is encoded by the gene protein tyrosine phosphatase non-receptor type 6 (*PTPN6*), is an essential component of the protein tyrosine phosphatase (*PTP*) family [18, 19]. *SHP-1* functions as a negative regulator in various cellular signalling pathways [19, 20], primarily by dephosphorylating tyrosine residues on various signalling proteins (such as signal transducer and activator of transcription (*STAT*), protein kinase B (*Akt*) and *Src*). This dephosphorylation modulates tumour, inflammatory, and metabolic pathways [21], inhibiting signal transduction by targeting tyrosines in proteins. These factors significantly influence various critical biological activities of related cells and are essential for maintaining normal cellular functions and the integrity of the immune system [18]. Although *SHP-1* is predominantly expressed in haematopoietic cells, it has also been identified in nonhaematopoietic cells, such as ECs [22]. Research indicates that bovine aortic ECs contain endogenous *SHP-1* [23]. Additionally, this molecule functions as a negative modulator, blocking superoxide generation in these cells [24]. In ECs under hypoxia, *SHP-1* inhibits reactive oxygen species (ROS) generation, reduces the stability of hypoxia-inducible factor 1-alpha (*HIF-1α*), and promotes the secretion of vascular endothelial growth factor (*VEGF*) to inhibit cell growth [25].

**Fig. 1.**
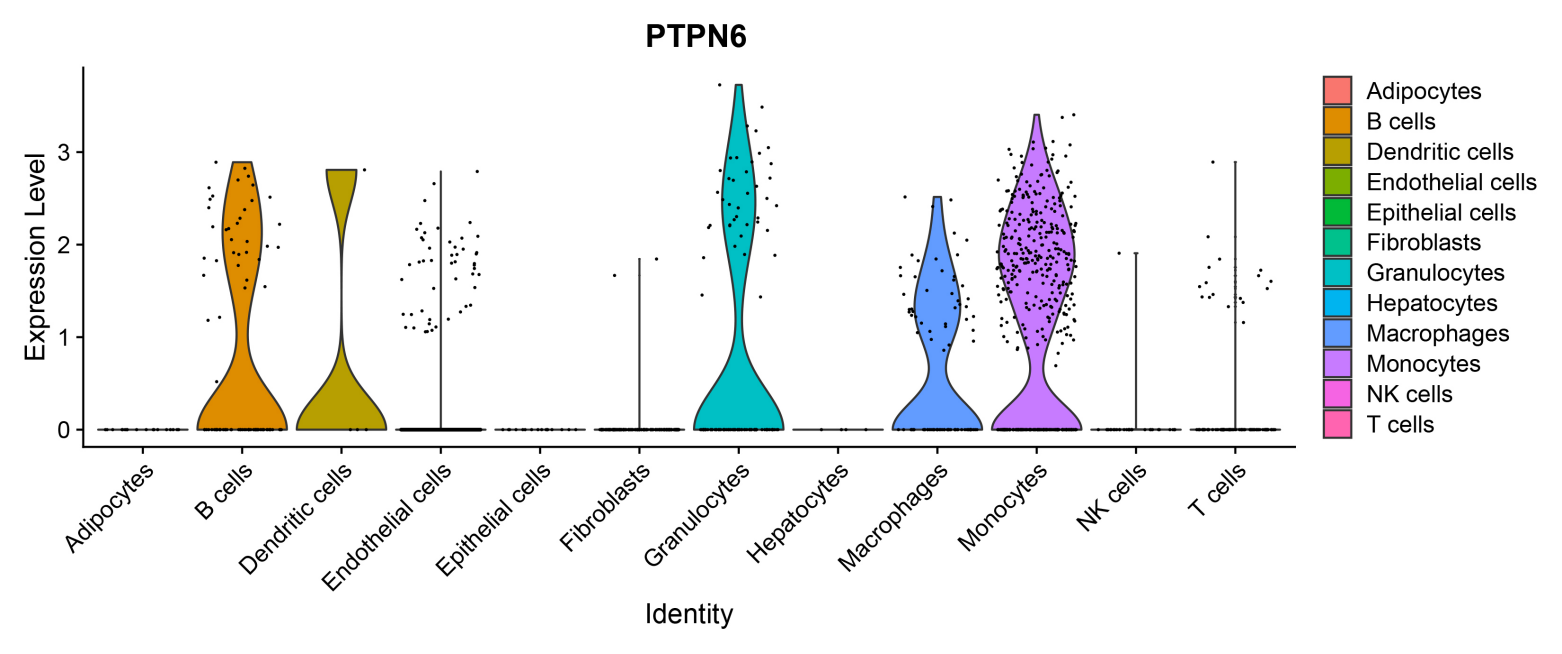
***SHP-1* expression in different cell subpopulations was analysed using the GEO database GSE154959 [26]**. *SHP-1*, Src homology region 2 domain-containing phosphatase-1; GEO, Gene Expression Omnibus; *NIK*, natural killer T cells, NKT cells; *PTPN6*, protein tyrosine phosphatase non-receptor type 6.

We analysed the cell-type specific expression of *SHP-1* based on single-cell RNA-seq data derived from the lung tissue samples of PH mice and retrieved from the GSE154959 dataset in the Gene Expression Omnibus (GEO) database. Specifically, *SHP-*1 is highly expressed in several immune cell populations, including macrophages, B cells, monocytes, granulocytes and dendritic cells. In contrast, *SHP-1* expression is significantly inhibited in ECs, whereas *SHP-1* expression is almost absent in nonimmune cells, such as adipocytes, epithelial cells, and hepatocytes (Fig. 1, Ref. [26]). This unique expression profile suggests that *SHP-1* is involved primarily in the regulation of immune cell functions. However, its low-level expression in ECs may indicate a specific role in vascular homeostasis or pulmonary vascular remodelling.

Therefore, the aim of this review is to explore the role and mechanisms of PAECs in the pathogenesis of PH. The possible mechanisms of endothelial *SHP-1* in PH-related pulmonary vascular remodelling are discussed in detail, which may provide novel therapeutic targets and insights for the prevention and treatment of PH.

## 2. Role and Mechanisms of PAECs in the Pathogenesis of PH

### 2.1 Roles of Genetic and Epigenetic Inheritance in PH

The genetic roles and mechanisms of PAECs in PH encompass multiple aspects, including abnormalities in transcription factors [27, 28, 29], mitochondrial dysfunction [30, 31], cell death pathways [30, 32], *EndMT* [33, 34], genetic variations, and metabolic abnormalities [34, 35]. Together, these mechanisms lead to PAEC dysfunction, which results in changes in the pulmonary vasculature and advances the progression of PH. Importantly, the most frequently identified genetic factor associated with familial PH is bone morphogenetic protein receptor type II (*BMPR2*) [35]. Some individuals with PH have a genetic predisposition, such as patients carrying a heterozygous abnormality in the gene encoding *BMPR2* and a mutation in the activin-like kinase (*Alk*)-1 receptor [5, 36, 37]. Mutant mice have increased susceptibility to hypoxia-induced PH, along with impaired endothelium-dependent vasodilation within the pulmonary vasculature [38]. Heterozygous *Bmpr2* knockout leads to EC injury and persistent PH in mice.

Epigenetic inheritance describes how gene expression can be altered without any modification to the DNA sequence itself. This mechanism is influenced by factors such as DNA methylation, histone modifications, and noncoding RNA [39]. A recent epigenome-wide association study (EWAS) revealed a total of 865,848 differentially methylated cytosine-phosphate-guanine (*CpG*) sites in the peripheral blood samples of patients suffering from PH [40], underscoring the widespread occurrence of epigenetic dysregulation. Within vascular ECs, modifications to histones are crucial for disease progression. The targeted inhibition of important elements within the histone H3 lysine 4 (*H3K4*) methyltransferase complex, specifically absent, small, or homeotic 2 (*ASH2*) and WD repeat-containing protein 5 (*WDR5*), significantly ameliorates hypoxia-induced PH in mice, confirming the critical role of histone methylation regulation [41].

Furthermore, studies utilizing the pulmonary thromboembolism (PTE) rat model have demonstrated that the miR-124/polypyrimidine tract binding protein 1 (*PTBP1*)/pyruvate kinase M (*PKM*) signalling axis facilitates pulmonary artery intimal remodelling through the mediation of metabolic reprogramming [42]. Collectively, these findings indicate that (1) epigenetic mechanisms, including DNA methylation and histone modifications, drive abnormal endothelial cell proliferation and vascular remodelling by regulating the expression of key genes and that (2) energy metabolism disorders resulting from metabolic reprogramming further exacerbate the pathological thickening of the pulmonary artery intima. These two mechanisms may be interrelated and jointly contribute to the progression of pulmonary hypertension.

### 2.2 Role of PAEC Dysfunction in PH

The primary trigger for PH is the dysfunction of PAECs, which is predominantly characterized by the generation of associated active factors and alterations in coagulation within the pulmonary endothelium. This dysfunction leads to abnormal contractions of the pulmonary vasculature, *in situ* thrombosis, and the remodelling of vascular structures, ultimately contributing to the onset and progression of PH. This condition represents an endothelial pathological state resulting from an imbalance between substances that induce contraction and those that promote vasodilation [43].

Studies have confirmed that PAEC dysfunction disrupts the pathological proliferation and migration of adjacent PASMCs, ultimately leading to thickening of the vascular wall’s medial layer and a progressive increase in pulmonary vascular resistance [44, 45].

Mice with defects in ECs and haematopoietic cells that encode prolyl-4 hydroxylase 2 (*PHD2*) exhibit severe occlusive vascular remodelling and right heart failure. In particular, the pulmonary vascular lesions of these mice significantly increased EC proliferation. Reactivation of hypoxia-inducible factor 2α (*HIF-2α*) signalling in ECs is a crucial factor in the development of PH. Endothelial *HIF-2α* activation is the primary mechanistic link for the development of PH after PHD2 deficiency [46, 47]. Additionally, a reduction in pulmonary endothelial *HIF-*2α causes a significant loss of hypoxia-induced PH in these mice [48]. Studies have shown that P-selectin and von Willebrand factor (*vWF*) are procoagulant factors located on pulmonary ECs. Increases in these factors reflect damage and dysfunction [49, 50].

The above studies elucidate the central role of EC dysfunction in the pathogenesis of PH. This dysfunction leads to pulmonary vasoconstriction, thrombus formation, and medial thickening through an imbalance of active factors, coagulation abnormalities, and *HIF-2α*-driven vascular remodelling, ultimately resulting in increased pulmonary vascular resistance and right heart failure.

### 2.3 Role of the Immune Inflammatory Response in PH

In PH, inflammation is characterized by (1) elevated levels of cytokines, chemokines, and adipokines and (2) varying degrees of inflammatory and immune cell infiltration surrounding and within the walls of small pulmonary arteries [51]. Furthermore, one study revealed the presence of tertiary lymphoid tissues (tLTs) in the lungs of patients with idiopathic pulmonary arterial hypertension (IPAH), which may be associated with aberrant immune system activation and autoantibody production [52]. Another study demonstrated that anti-endothelial cell antibodies (*AECAs*) are detectable in patients with systemic sclerosis (SSc) and are correlated with a greater incidence of vascular lesions and related symptoms. *AECAs* can activate ECs and lead to apoptosis in patients. Additionally, PH can also occur in patients, contributing to increased mortality [53]. A study conducted by Sasaki N’s team [54] suggested that the induction of PAEC apoptosis by a combination of anti-endothelial cell antibodies and activated natural killer cells may play a crucial role in the vascular damage associated with PH in patients with mixed connective tissue disease. These findings underscore the significant role of the immune system in PH.

### 2.4 Role of Oxidative Stress in PH

Oxidative stress is recognized as a critical factor leading to EC injury and functional impairment [55]. Increases in ROS production lead to an imbalance in the signalling between reactive nitrogen species (RNS) and nitric oxide (NO) [56], as well as to DNA damage [57]. This imbalance results in abnormal proliferation, injury, and apoptosis of ECs. Oxidative stress can also promote the development of PH by disrupting the NO signalling pathway. As a crucial vasodilatory mediator, the synthesis of NO is regulated primarily by endothelial nitric oxide synthase (*eNOS*). Under pathological conditions associated with PH, dysfunction of *eNOS* leads to its uncoupling, subsequently disrupting NO signalling and significantly impairing the capacity for vasodilation. This pathological alteration not only exacerbates vasoconstrictive responses but also further accelerates the progression of pulmonary arterial hypertension [56].

### 2.5 Role of Autophagy in PH

Research has demonstrated that oestradiol directly inhibits the proliferation of ECs and improves haemodynamics. By enhancing mitochondrial autophagy, oestradiol also inhibits PH [58]. Conversely, it enhances EC angiogenesis in foetal lambs with persistent PH, which reduces the expression of the autophagy protein beclin-1, leading to autophagy defects [59]. Moreover, autophagy accelerates the transition from an apoptotic phenotype to a hyperproliferative phenotype in pulmonary vascular ECs associated with HIV-related PH [60].

Singh *et al*. [61] reported increased expression and activity of fatty acid synthase (*FAS*) in hypoxic human pulmonary artery smooth muscle cells (HPASMCs). The inhibition of FAS promotes HPASMC apoptosis and reduces autophagy, which reduces pulmonary vascular remodelling and endothelial dysfunction [61].

### 2.6 Role of EndMT in PH

*EndMT* induced by dysfunctional PAECs is considered the initial step and a key pathological factor in the occurrence of PH [62]. In PH, *EndMT* directly promotes structural changes in the vascular wall by causing PAECs to lose their endothelial characteristics and acquire the migratory and proliferative abilities of mesenchymal cells [33, 62, 63]. In PH associated with congenital heart disease, high shear stress (HSS) can directly induce *EndMT*, thereby initiating vascular remodelling [64]. Numerous studies have demonstrated that apoptosis, inflammation, and metabolic abnormalities, such as oxidative stress in PAECs, can induce *EndMT*. These findings suggest that *EndMT* may serve as a compensatory response following endothelial injury [65]. This dual regulatory role positions *SHP-1* as a pivotal therapeutic target for modulating vascular remodelling processes [66].

## 3. The Expression and Regulation of *SHP-1* in eECs

### 3.1 Expression Characteristics of SHP-1 in ECs

Studies have demonstrated that *SHP-1* is expressed in various epithelial tissues, including haematopoietic cells and ECs [24, 67]; however, its expression level in ECs remains relatively low. In human microvascular endothelial cells (HMECs), *SHP-1* is predominantly localized in the nucleus, with only moderate expression observed in the cytoplasm [25].

### 3.2 Core Role of SHP-1 in Vascular Homeostasis and Disease

*SHP-1* plays a critical role in ECs by protecting them from the upregulation of adhesion molecules and the harmful effects of thrombosis under inflammatory conditions. Under hypoxic or ischaemic conditions, *SHP-1* promotes the development of blood vessels by suppressing oxidative stress. In ischaemic illnesses, *SHP-1* suppresses the production of ROS, which in turn inhibits the proliferation and survival of ECs [25].

For example, in a diabetic mouse model, hyperglycaemia impairs the vascular regenerative capacity of ischaemic muscles by upregulating *SHP-1* expression in ECs, which inhibits the activity of angiogenic factors [68]. In an *in vitro* model of chronic obstructive pulmonary disease (COPD), the expression level of *SHP-1* was significantly decreased. *SHP-1* overexpression reversed the effects of cigarette smoke extract (CSE) on endothelial cell migration, epithelial‒mesenchymal transition (*EMT*), and the production of proinflammatory factors. Moreover, it mitigated the inflammatory response by inhibiting the P65 and *PI3K/AKT* signalling pathways [69]. In the diabetic state, *SHP-1* promotes endothelial cell senescence and contributes to abnormal collateral vessel formation by diminishing the proangiogenic effects of nuclear factor erythroid 2-related factor 2 (*Nrf2*) and *VEGF*, ultimately impeding blood flow reperfusion. However, the overexpression of dominant-negative *SHP-1* (dn*SHP-1*) effectively reverses these pathological effects [62]. In diabetic peripheral arterial disease, *SHP-1* reduces endothelial cell migration and capillary formation by negatively regulating the vascular endothelial growth factor receptor 2 (VEGFR2) and platelet derived growth factor receptor beta (*PDGFR-β*) signalling pathways [70].

Comprehensive evidence indicates that maintaining moderately high *SHP-1* expression is crucial for controlling inflammation and ensuring endothelial homeostasis. The lack of expression of this molecule has emerged as a common pathological feature in various vascular diseases. These findings underscore the importance of *SHP-1* as a vital target for research in the context of vascular diseases and inflammatory responses.

## 4. The Key Signalling Pathway That Regulates the Expression of *SHP-1*

### 4.1 SHP-1 Regulates the Phosphorylation Level of VEGFR2

#### 4.1.1 Target Action

*SHP-1* can indirectly affect the phosphorylation of *VEGF*R2 by dephosphorylating Src family kinases (such as *Lyn* and *Fyn*) [71]. This dephosphorylation depends on the interaction between *SHP-1* and the *SH2* domain of Src family receptors [72]. Upon *VEGF* stimulation, the phosphatase activity of *SHP-1* is activated, leading to the dephosphorylation of specific tyrosine residues (such as Y996, Y1059, and Y1175) on *VEGF*R2, thereby inhibiting *VEGF*R2 signalling. This dephosphorylation attenuates *VEGF*R2-mediated downstream signalling pathways, such as the activation of extracellular signal-regulated kinase (*ERK*) and *Akt*, consequently suppressing the proliferation and DNA synthesis of vascular ECs [71]. Cellular communication network factor 1 (*CCN1*), also known as cysteine-rich protein sixty-one, is a stromal cell protein that interacts with integrins and is secreted by the cell. Cardiovascular system development is highly important in human life.* CCN1* enhances *SHP-1* activity by binding to *VEGF*R2, leading to *VEGF*R2 dephosphorylation and the inhibition of endothelial cell proliferation [73].

#### 4.1.2 Evidence of Inhibiting Angiogenesis Through the Regulation of SHP-1

Furthermore, this study revealed that acetyl-11-keto-boswellic acid (*AKBA*) can upregulate the expression and activity of *SHP-1*. The upregulation of *SHP-1* by *AKBA* leads to reduced *VEGF* expression and downregulated phosphorylation of *VEGF*R2 and *STAT3*, thus inhibiting angiogenesis. Overall, these findings underscore the critical role of *SHP-1* in regulating endothelial cell angiogenesis [74].

#### 4.1.3 Evidence to Support That Regulating SHP-1 Inhibits Vascular Permeability

Clearly distinguishing the mechanisms underlying changes in vascular permeability between PH and acute lung injury (ALI)/acute respiratory distress syndrome (ARDS) is crucial. Both ALI and ARDS are marked by a breakdown of the alveolar-capillary barrier, which is evident through a strong inflammatory reaction that causes damage to both endothelial and alveolar epithelial cells injury, ultimately culminating in the accumulation of protein-rich pulmonary edema [75]. ARDS signifies the critical phase of ALI, marked by significant formation of hyaline membranes, collapse of alveoli, and persistent hypoxemia [76, 77]. Conversely, the changes in vascular permeability seen in PH mainly stem from endothelial dysfunction that occurs throughout the chronic remodeling of the pulmonary vasculature. This condition presents as perivascular edema rather than as exudation into the alveolar spaces, and the underlying mechanisms differ fundamentally from the acute inflammatory damage seen in ALI/ARDS [78].

Cytokine TNF superfamily member 15 (*TNFSF15*) is produced primarily by vascular ECs, and receptor activation leads to trimerization with *VEGF*R2 and death receptor 3 (*DR3*). This process affects the activity of *SHP-1* phosphatase, which further inhibits the phosphorylation of *VEGF*R2 [79]. In addition, Chu* et al*. [80] reported that thrombospondin-1 (*TSP-1*) binds to *VEGF*R2 via its interaction with *STAT3* while recruiting *SHP-1* to inhibit the phosphorylation of *VEGF*R2; thus, TSP-1 reduces the phosphorylation level of *VEGF*R2 and *VEGF*-induced endothelial cell migration. The thrombospondin type 1 repeat (*TSR*) domain inhibits tube formation [80].

Additionally, *SHP-1* phosphatase activity is enhanced by a novel aliphatic isohydroxamic acid ester derivative (*WMJ-S-001*), resulting in the inhibition of *VEGF*R2 phosphorylation within the *VEGF-A-VEGFR2* signalling pathway, which ultimately decreases the cytogenic activity of vascular ECs [81].

#### 4.1.4 Role of SHP-1 in the Pathological Environment

Studies have shown that hyperglycaemic and hypoxic environments upregulate the phosphatase activity of *SHP-1*, which inhibits *VEGF* signalling. This process impairs the functional ability of ECs and inhibits angiogenesis [25, 82, 83]. In addition, N(ε)-carboxymethyl lysine (CML) activates ROS signalling via NADPH oxidase, which in turn enhances *SHP-1* activity. Consequently, *SHP-1* damages ECs by dephosphorylating *VEGF*R-2, which results in EC dysfunction [84].

#### 4.1.5 Gene Intervention and Animal Model Validation

In an *in vitro* study, *SHP-1* inhibition promoted the ability of TNF-α to impede the *VEGF*-driven phosphorylation of *VEGF*R-2, which promoted the growth of ECs. In a rat model of hind limb ischaemia, the expression levels of *SHP-1* and *VEGF* were elevated *in vivo*. Treatment with siRNA that suppressed *SHP-1* gene expression significantly increased both *VEGF*R-2 phosphorylation and capillary density, indicating that *SHP-1* is a negative regulator of angiogenesis [85]. Treatment with *VEGF* resulted in the c-Src kinase-dependent activation of *SHP-1* phosphatase activity. Inhibition of *SHP-*1 with *siRNA* or *c-Src* results in elevated tyrosine phosphorylation levels of *VEGF*R-2 and phosphorylation extracellular signal-regulated kinase (*pERK*), which enhances DNA synthesis and promotes EC proliferation. These results show that *SHP-1* is essential for the regulation of ECs [80].

### 4.2 SHP-1 Regulates the JAK2/STAT3 Signalling Pathway

#### 4.2.1 Target Action

As a tyrosine phosphatase with an SH2 domain, *SHP-1* can directly bind to *JAK2* and dephosphorylate its substrate *STAT3*. As a result, it negatively regulates the activation of the *JAK*/*STAT3* signalling pathway, which helps maintain ECs under normal conditions [86, 87].

#### 4.2.2 Abnormal Regulation of SHP-1 Under Pathological Conditions

The levels and activity of *SHP-1* control critical functions of ECs, such as proliferation, migration, and angiogenesis. For example, in a high-glucose environment, the inhibition of *SHP-1* activity can result in hyperactivation of the *JAK*/*STAT3* signalling pathway, leading to abnormal endothelial cell injury and angiogenesis [88]. Additionally, the expression and activity of *SHP-1* are modulated by various factors. Under certain pathological conditions, *SHP-1* expression may be downregulated, or its activity may be suppressed, resulting in the aberrant activation of the *JAK/STAT3* signalling pathway [89, 90, 91].

#### 4.2.3 Pharmacological Intervention and Therapeutic Potential

This study revealed that naringenin can inhibit *JAK2/STAT3* signalling pathway activation while increasing the expression of *SHP-1*, which improved hypertension during pregnancy. Sufficient evidence indicates that *SHP-1* must be expressed and activated to suppress oxidative stress, inflammatory responses, and *JAK2/STAT3* signalling pathway activity. This alleviation of damage to vascular endothelial cell damage and vasoconstriction further regulates the development and differentiation of ECs [92]. Angiopoietin 1 (*Ang1*) inhibits cell proliferation; in this context, the induction of *SHP-1* dampens Ang1-mediated interleukin 6 (*IL-6*)-induced stimulation of the* JAK/STAT3* signalling pathway, thus reducing *IL-6*-induced endothelial cell permeability and suppressing the vascular immune‒inflammatory response [93]. Moreover, some studies have shown that inhibiting Phloretin activates *SHP-1* to phosphorylate *STAT3*. This process can ultimately induce apoptosis and autophagy in vascular ECs [94, 95].

### 4.3 SHP-1 Regulates ERK Phosphorylation

#### 4.3.1 Target Action

*SHP-1* can bind to epidermal growth factor receptor (*EGFR*) and dephosphorylate its downstream substrates, thereby suppressing EGFR-mediated *ERK* activation [96]. *SHP-1* suppresses angiogenesis and inflammatory responses through the dephosphorylation of key signalling molecules, such as *ERK* and c-Jun N-terminal kinase (*JNK*) [97, 98].

#### 4.3.2 Functional Verification

Stimulation of bovine aortic ECs with *VEGF* and epidermal growth factor (*EGF*) significantly increased the phosphorylation of *ERK*. However, treatment with *TNF-α* for 10 minutes attenuated the phosphorylation of *ERK*. Importantly, the overexpression of *SHP-1* effectively prevented the inhibition of *ERK* phosphorylation induced by *TNF-α*. *TNF-α* blocks the growth factor-induced phosphorylation of *ERK*-induced EC proliferation by activating *SHP-1*. The activation of *SHP-1* suppresses the phosphorylation of *E23RK *induced by growth factors, including *VEGF*, EGF, and platelet-derived growth factor (*PDGF*). Endothelial cell proliferation, differentiation, and transformation are downregulated [23, 70].

#### 4.3.3 Animal Model Validation

Knockout of the connexin 37 (*Cx37*) gene in mice might increase *SHP-1* activity, which in turn could lead to the dephosphorylation of proteins such as myosin light chain 2 (*MLC2*),* ERK*, and protein kinase B through various mechanisms. The angiotensin II (*Ang II*) signalling cascade involves the phosphorylation of proteins generated after the activation of *Ang II* at the AT1 receptor (*AT1R*) in ECs. Protein dephosphorylation may interfere with important cellular physiological processes, such as contraction, proliferation, and survival [99].

### 4.4 SHP-1 is Involved in Regulating the Levels of ROS and HIF-1

#### 4.4.1 The Oxygen-dependent Regulatory Mechanism of HIF-1α

*HIF-1* consists of an oxygen-regulated α subunit and a constitutively expressed β subunit [100, 101]. Under normoxia, prolyl hydroxylase facilitates the degradation of *HIF-1α*, whereas hypoxia blocks this process, leading to the stabilization and accumulation of *HIF-1α* [102, 103]. *HIF-1α* is an important transcription factor under low-oxygen conditions. It can regulate the production of *VEGF* [104, 105].

#### 4.4.2 Regulation of the ROS/HIF-1α/VEGF Axis by SHP-1

Under hypoxic conditions, *SHP-1* knockdown increases ROS in ECs and further induces ROS to upregulate the expression of the *HIF-1α* protein [25]. The synthesis and production of *VEGF* are increased when SHP-1 is knocked down. Moreover, *SHP-1* also negatively regulates the production of ROS. ROS increase the stability of *HIF-1α*by inhibiting the enzyme activity of prolyl hydroxylase, leading to its degradation [106]. Thus, *SHP-1* knockdown leads to an increase in ROS, which stabilizes *HIF-1α*. In summary, *SHP-*1 regulates cell proliferation and *VEGF* synthesis by altering the *HIF-1α* and ROS levels [25].

### 4.5 Other Avenues

Under hyperglycaemic conditions, *SHP-1* is activated and binds to DR3, the receptor for tumour necrosis factor ligand-related molecule 1A (*TL1A*). This activation inhibits the dephosphorylation of Src by *SHP-1*. Consequently, when glucose levels are elevated, *SHP-1* binds to the receptor of *TL1A*, also called death receptor 3 (*DR3*). This action of *SHP-1* prevents the dephosphorylation of Src, which then activates vascular endothelial-cadherin (*VE-cadherin*). As a result, EC integrity is impaired, leading to vascular leakage [107].

*SHP-1* plays a crucial role in maintaining vascular haemostasis within the body. During the inflammation of ECs caused by *TNF-α*, *SHP-1* inhibition enhances interactions between platelets and ECs, ultimately leading to arterial thrombosis. This autoinhibitory feedback mechanism of phosphatases is believed to prevent excessive inflammation and thrombosis [108]. *TNF-α* inhibits *VEGF*- and EGF-stimulated EC proliferation via *SHP-1* activation. Under hypoxia, blocking tumor necrosis factor receptor 1 (*TNFR-1*) or *SHP-1* in human umbilical vein endothelial cells (HUVECs) upregulated the expression of proangiogenic genes (*VEGF*R2 and *eNOS*) and a prosurvival gene (*Bcl-xL*) while downregulating the expression of a proapoptotic gene (*Bax*). Inhibiting *TNFR-1* or *SHP-1* with siRNA leads to increased HUVEC growth and differentiation [109].

Many findings present strong evidence that *SHP-1* negatively regulates endothelial cell function via tyrosine phosphatase activity. The activation of *SHP-1* inhibits the coagulant activity of ECs; however, loss of function or expression is able to attenuate this effect (Table 1, Ref. [23, 24, 25, 70, 73, 74, 79, 80, 81, 82, 83, 84, 85, 88, 92, 93, 94, 95, 97, 98, 99, 107, 108, 109, 110]).

**Table 1.** **Differential regulation of endothelial cell function by *SHP-1* activity status**.

Inhibits phosphorylation levels of *VEGF*R2				
*SHP-1* activation	Nature of study	Regulation	EC dysfunction	Reference
↑	*In vitro*, *in vivo*	–	Proliferation	[73]
↑	*In vitro*, *in vivo*	–	Vascular permeability	[79]
↑	*In vitro*, *in vivo*	–	Angiogenesis	[74]
↑	*In vitro*, *in vivo*	–	Angiogenesis	[81]
↑	*In vitro*, *in vivo*	–	Migrate	[80]
↑	*In vitro*, *in vivo*	–	Angiogenesis	[25, 82, 83]
↑	*In vitro*, *in vivo*	–	Oxidative stress	[84]
↓	*In vitro*, *in vivo*	+	Angiogenesis	[85]
↓	*In vitro*	+	Proliferation	[110]
Inhibits the* JAK2/STAT3* signalling pathways				
*SHP-1* activation	Nature of study	Regulation	EC dysfunction	Reference
↓	*In vitro*	+	Angiogenesis	[88]
			Endothelial cell injury	
↑	*In vivo*	–	Growth	[92]
↑	*In vitro*	–	Immune inflammation	[93]
↑	*In vitro*, *in vivo*	–	Apoptosis, autophagy	[94, 95]
Inhibits *ERK* phosphorylation				
*SHP-1* activation	Nature of study	Regulation	EC dysfunction	Reference
↑	*In vitro*, *in vivo*	–	Angiogenesis	[97]
			inflammation	[98]
↑	*In vitro*, *in vivo*	–	Proliferation	[23, 70]
↑	*In vivo*	–	Proliferation	[99]
*SHP-1* is involved in regulating the levels of ROS and *HIF-1*				
*SHP-1* activation	Nature of study	Regulation	EC dysfunction	Reference
↑	*In vivo*	–	Proliferation	[25]
Other avenues				
*SHP-1* activation	Nature of study	Regulation	EC dysfunction	Reference
↑	*In vitro*, *in vivo*	–	Immune inflammation	[107]
↑	*In vivo*	–	inflammation	[24]
↑	*In vitro*	–	Oxidative stress	[108]
↑	*In vitro*	–	Proliferation	[23]
↓	*In vitro*	+	Proliferation	[109]

Signals may initiate (↑) or inhibit (↓) SHP-1 activity. Positively (+) or negatively (–) regulate endothelial cell (EC) functions. *SHP-1*, Src homology region 2 domain-containing phosphatase-1; *VEGFR2*, vascular endothelial growth factor receptor 2; EC, endothelial cell; *JAK2*, janus kinase 2; *STAT3*, signal transducer and activator of transcription 3; *ERK*, extracellular signal-regulated kinase; ROS, reactive oxygen species; *HIF-1*, hypoxia-inducible factor 1.

## 5. Potential Mechanism of Endothelial *SHP-1* in PH-Related Pulmonary Vascular Remodelling

### 5.1 Suppression of the Proliferation, Migration and Angiogenesis of ECs

*SHP-1* plays a crucial role in regulating EC function and angiogenesis by modulating the phosphorylation of *VEGF*R2 and its downstream signalling pathways. Molecules such as* CCN1*, limonin, *AKBA*, and *WMJ-S-001* activate *SHP-1* and inhibit *VEGF*R2 phosphorylation, thereby suppressing endothelial cell proliferation, migration, and angiogenesis [73, 74, 81]. Under conditions such as hyperglycaemia and hypoxia, the activity of *SHP-1* is increased. By suppressing the overactivation of the *VEGF* and JAK/*STAT3* signalling pathways, *SHP-1* contributes to endothelial cell dysfunction and impaired angiogenesis [25, 82, 83]. Furthermore, the inhibition of *SHP-1* in HUVECs promotes *VEGF*R2 expression and drives endothelial cell proliferation [109]. The activation of *SHP-1* induces dephosphorylation of the *ERK* protein, thereby modulating endothelial cell proliferation [23, 70, 99]. Under hypoxic conditions, *SHP-1* regulates endothelial cell proliferation by controlling *HIF-1α* and ROS levels [25, 102, 103]. Collectively, these studies demonstrate that the deletion or reduction of *SHP-1* leads to excessive endothelial cell proliferation and migration as well as abnormal vascular formation.

### 5.2 Regulating Endothelial Apoptosis and Autophagy

Phloretin inhibits the phosphorylation of *STAT3* by activating *SHP-1*, which induces apoptosis and autophagy in ECs [94, 95]. Under hypoxic conditions, the inhibition of *TNFR-1* or *SHP-1* in HUVECs significantly increases the expression of the antiapoptotic factor *Bcl-xL* while decreasing the expression of the proapoptotic factor *Bax*, thereby effectively suppressing apoptosis in these cells [109].

### 5.3 Regulating the Inflammatory Response

*SHP-1* suppresses angiogenesis and inflammatory responses by dephosphorylating key signalling molecules, including *ERK* and *JNK* [97, 98]. Studies have shown that Ang1 activates *SHP-1* to inhibit IL-6-induced endothelial cell permeability and inflammatory responses [108]. Furthermore, the inhibition of *SHP-1* exacerbates *TNF-α*-induced endothelial inflammation, while its self-inhibitory feedback mechanism serves to prevent excessive inflammatory activation [108].

### 5.4 Regulation of Vascular Permeability

Under hyperglycaemic conditions, *SHP-1* is activated and binds to DR3, impairing its ability to dephosphorylate Src. This process induces the activation of *VE-cadherin*, which destabilizes endothelial cell integrity and contributes to vascular leakage [107].

### 5.5 Regulation of the Oxidative Stress Response

In HUVECs, *SHP-1* negatively regulates Rac1 activation by suppressing PI3K activity, thereby modulating NAD(P)H oxidase-dependent superoxide production and significantly reducing oxidative stress levels in ECs [24].

### 5.6 Summary

**Fig. 2.**
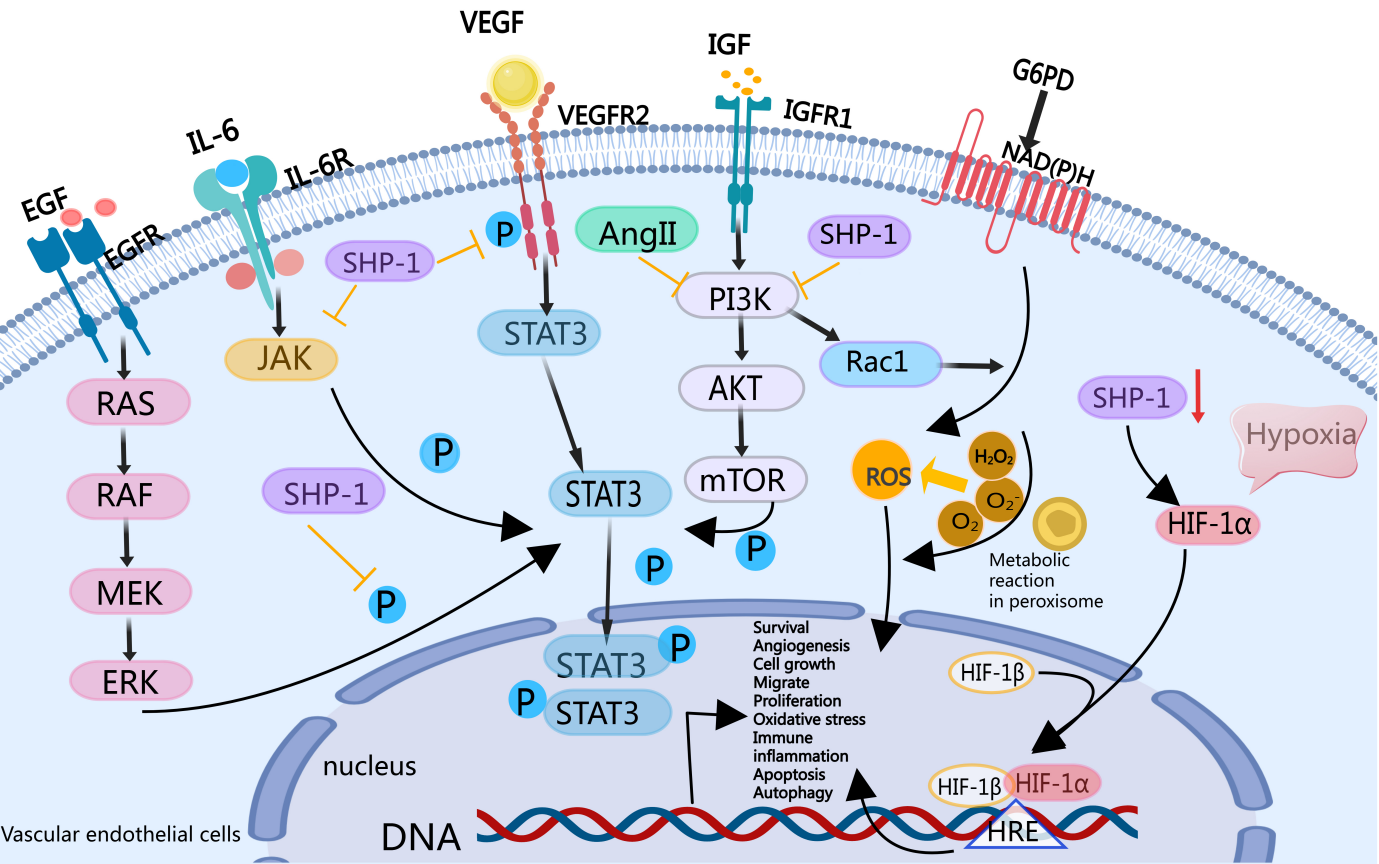
***SHP-1* may regulate PAEC function to inhibit signalling pathways that result in PH vascular remodelling**. *SHP-1* inhibits *VEGF*R2/IGFR1, the *EGFR/ERK*-mediated *STAT3* signalling pathway, and the *IL-6*-induced JAK/*STAT3* signalling pathway; and the *PI3K/Rac1* signalling pathway negatively regulates NADPH oxidase (*NOX*)*/*vascular peroxidase 1 (*VPO1*) pathway-derived ROS. *SHP-1* inhibits EC migration and proliferation, immune inflammation, activation, oxidative stress, vasoconstriction, generation, vascular permeability, apoptosis, autophagy, and other important pathophysiological processes in the development of PH. Additionally, the silencing of *SHP-1* leads to an increase in ROS and hypoxia-inducible factor 1 alpha (*HIF-1α*), which may further increase the proliferation of ECs and influence pulmonary vascular remodelling in PH. Created with MedPeer (medpeer.cn). HRE, hypoxia response element; PAEC, pulmonary artery endothelial cell; PH, pulmonary hypertension; *VEGF*R2, vascular endothelial growth factor receptor 2; *EGFR*, epidermal growth factor receptor;* ERK*, extracellular signal-regulated kinase; *IL-6*, interleukin 6; *PI3K*, Phosphoinositide 3-Kinase; ROS, reactive oxygen species; EC, endothelial cell; EGF, epidermal growth factor; *STAT3*, signal transducer and activator 0f Transcription 3; *JAK*, janus kinase; *Rac1*, rac family small GTPase 1; *NADPH*, nicotinamide adenine dinucleotide phosphate; *IGFR1*, insulin-like growth factor receptor 1; *G6PD*, glucose-6-phosphate dehydrogenase; *Ang II*, angiotensin II; *RAS*, aenin-angiotensin system; *RAF*, rapidly accelerated fibrosarcoma; *MEK*, *MAPK/ERK* kinase.

In summary, endothelial *SHP-1* may suppress the development and progression of PH through multiple mechanisms. Specifically, *SHP-1* inhibits EC migration and proliferation, reducing key drivers of vascular remodelling; *SHP-1* modulates immune-inflammatory responses and oxidative stress to mitigate endothelial cell damage; *SHP-1* suppresses pathological angiogenesis by inhibiting signalling pathways such as *VEGF*; and *SHP-1* regulates vascular permeability while inhibiting apoptosis and autophagy to maintain endothelial cell function and stability. These combined effects inhibit pulmonary vascular remodelling and prevent the progression of PH, highlighting *SHP-1* as a potential therapeutic target for PH treatment (Fig. 2).

## 6. Therapeutic Prospects of SHP-1 Activators/Inhibitors in Vascular Diseases

### 6.1 Therapeutic Potential of SHP-1 Activators

The SHP-1 activators exert antiproliferative and pro-apoptotic effects by inhibiting B cell receptor (*BCR*) signalling pathway, as evidenced by the downregulation of p-Lyn. This inhibition may also indirectly influence tumour angiogenesis [111, 112]. The overexpression of SHP-1 counteracts the migration of endothelial cells and the release of inflammatory factors triggered by CSE, indicating that SHP-1 has a protective function in chronic inflammatory vascular conditions, including vascular lesions associated with COPD [69]. Angiogenesis is influenced by SHP-1 through the regulation of TGF-β1 signalling, potentially facilitating the development of collateral circulation in models of ischaemia [113].

### 6.2 Potential Risks of SHP-1 Inhibitors

In certain types of cancer, the activation of SHP-1 may foster a microenvironment that is favourable for tumours, indicating that caution is warranted when using SHP-1 inhibitors to treat vascular-related tumours [87]. SHP-1 suppresses overly active immune responses within Treg cells; nonetheless, a lack of this protein might result in T-cell impairment, which can influence the development of vascular autoimmune disorders [114].

### 6.3 Summary

Modulators of SHP-1 have the potential to play dual roles in vascular disease treatment: they may enhance the healing of atherosclerotic or diabetic vascular lesions by providing anti-inflammatory benefits and protecting endothelial cells as an activator, whereas they may facilitate the regeneration of blood vessels in ischaemic tissues under certain circumstances as an inhibitor. Additional research is essential to clarify the mechanisms specific to different tissues and explore avenues for clinical application.

## 7. Conclusion and Clinical Implications

On the basis of current knowledge, *SHP-1* is suspected to play a significant role in the development and maintenance of pulmonary endothelial dysfunction associated with PH, potentially offering new therapeutic innovations for this condition. Several experiments could be conducted to test this hypothesis, including *in situ* studies on tissues from patients with and without PH to verify the expression and localization of the *SHP-1* protein in various cell types within remodelled pulmonary artery walls. Because PH is typically diagnosed at advanced stages and patients are often treated with multiple therapeutic agents, tracking *SHP-1* expression and its targets in preclinical models of PH at different stages of its development is crucial. Such analyses include using the chronic Sugen-hypoxia model, models severe PH induced by monocrotaline, and the combination model in rats. Furthermore, *in vitro* experiments could be designed to investigate the molecular mechanisms regulated by *SHP-1* in dysfunctional PAECs from PH patients by manipulating *SHP-1* expression levels in PAECs from patients without PH. Using haemodynamic data from adult knockout or conditionally overexpressing *SHP-1* mice or rats and evaluating the efficacy of *SHP-1* agonist treatments in preclinical models are both essential to strengthening these observations. In these *in vivo* studies, it will be important to thoroughly assess cardiac function to ensure that these approaches do not negatively impact ventricular performance, including adaptive hypertrophy of the right ventricle. Taken together, these data could be used to determine whether restoring *SHP-1* expression is a promising novel intervention in the treatment of PH.

## Data Availability

The datasets [ANALYZED] for this study can be found in the [Gene Expression Omnibus] [https://www.ncbi.nlm.nih.gov/geo/query/acc.cgi?acc=GSE154959].

## Abbreviations

PH, pulmonary hypertension; PAECs, pulmonary artery endothelial cells; SHP-1, Src homology region 2 domain-containing phosphatase-1; mPAP, mean pulmonary artery pressure; EMT, endothelial mesenchymal transition; PTP, protein tyrosine phosphatase; GEO, Gene Expression Omnibus; ECs, endothelial cells; BMPR2, type II bone morphogenetic protein receptor; Alk, activin-like kinase; ASH2, absent, small, or homeotic 2; WDR5, WD repeat-containing protein 5; VEGF, vascular endothelial growth factor; PHD2, prolyl-4 hydroxylase 2; Cas, Crk-associated substrate; VWF, von Willebrand factor; IL-6, interleukin 6; JNK, c-Jun N-terminal kinase; NOX, NADPH oxidase; VPO1, vascular peroxidase 1; FAS, fatty acid synthase; HPASMCs, human pulmonary artery smooth muscle cells; EndMT, endothelial-mesenchymal transition; siRNA, small-interfering RNA; HIF-2α, hypoxia-inducible factor 2α; CCN1, cellular communication network factor 1; TNFSF15, TNF superfamily member 15; DR3, death receptor 3; AKBA, acetyl-11-keto-b-boswellic acid; CML, carboxymethyl lysine; Ang1, angiopoietin 1; EGF, epidermal growth factor; ERK, extracellular signal-regulated kinase; PDGF, platelet-derived growth factor; Cx37, connexin 37; VE-cadherin, vascular endothelial cadherin; PI3K, phosphoinositide 3-Kinase; NO, nitric oxide; HUVECs, human umbilical vein endothelial cells; ALI, acute lung injury; ARDS, acute respiratory distress syndrome.
